# Carbapenem Breakpoints for *Acinetobacter baumannii* Group: Supporting Clinical Outcome Data from Patients with Bacteremia

**DOI:** 10.1371/journal.pone.0163271

**Published:** 2016-09-19

**Authors:** Yi-Tzu Lee, Mei-Chun Chiang, Shu-Chen Kuo, Yung-Chih Wang, I-Hsin Lee, Te-Li Chen, Ya-Sung Yang

**Affiliations:** 1 Faculty of Medicine, School of Medicine, National Yang-Ming University, Taipei, Taiwan; 2 Department of Emergency Medicine, Taipei Veterans General Hospital, Taipei, Taiwan; 3 Division of Preventive Medicine, Institute of Public Health, School of Medicine, National Yang-Ming University, Taipei, Taiwan; 4 National Institute of Infectious Diseases and Vaccinology, National Health Research Institute, Maoli County, Taiwan; 5 Division of Infectious Diseases and Tropical Medicine, Department of Internal Medicine, Tri-Service General Hospital, National Defense Medical Center, Taipei, Taiwan; 6 Institute of Clinical Medicine, School of Medicine, National Yang-Ming University, Taipei, Taiwan; 7 Institute of Biomedical Informatics, School of Medicine, National Yang-Ming University, Taipei, Taiwan; 8 Graduate Institute of Life Sciences, National Defense Medical Center, Taipei, Taiwan; 9 Division of Infectious Diseases, Department of Medicine, Taipei Veterans General Hospital, Taipei, Taiwan; Ross University School of Veterinary Medicine, SAINT KITTS AND NEVIS

## Abstract

The carbapenem breakpoints set by different organizations for *Acinetobacter* are discordant, but supporting clinical data are lacking. This study aimed to provide the first clinical outcome data to support the carbapenem breakpoints for *Acinetobacter baumannii* (Ab) group in patients with bacteremia. This study included 117 adults who received carbapenems for treatment of Ab group bacteremia in Taipei Veterans General Hospital over an 8-year period. We analyzed 30-day mortality rates among patient groups acquiring isolates with different carbapenem minimal inhibitory concentrations (MICs). The carbapenem MIC breakpoint derived from classification and regression tree (CART) analysis to delineate the risk of 30-day mortality was between MICs of ≤ 4 mg/L and ≥ 8 mg/L. Mortality rate was higher in patients acquiring isolates with carbapenem MIC ≥ 8 mg/L than ≤ 4 mg/L, by bivariate (54.9% [28/51] vs 25.8% [17/66]; *P* = 0.003) and survival analysis (*P* = 0.001 by log-rank test). Multivariate analysis using logistic regression and Cox regression models including severity of illness indices demonstrated that treating patients with Ab group bacteremia caused by isolates with a carbapenem MIC ≥ 8 mg/L with carbapenem was an independent predictor of 30-day mortality (odds ratio, 5.125; 95% confidence interval [CI], 1.946–13.498; *P* = 0.001, and hazard ratio, 2.630; 95% CI, 1.431–4.834; *P* = 0.002, respectively). The clinical outcome data confirmed that isolates with MIC ≤ 4 mg/L were susceptible to carbapenem, and those with MIC ≥ 8 mg/L were resistant in patients with Ab group bacteremia.

## Introduction

The phenotypically indistinguishable *Acinetobacter baumannii*, *Acinetobacter nosocomialis* and *Acinetobacter pittii* are grouped as the *A*. *baumannii* (Ab) group and have become major nosocomial pathogens associated with high mortality in immunocompromised hosts [1]. There is growing concern that Ab group is increasingly resistant to carbapenems [2–4], which are among the few antimicrobials that are still effective against these bacteria.

The selection of antimicrobial therapy is mainly determined by pathogen susceptibility. Therefore, a susceptibility breakpoint that correlates well with the clinical outcome is crucial. Breakpoints set at an erroneously high level might lead to prescription of incorrect antimicrobials, which can have a serious outcome in patients with Ab group bacteremia [5, 6]. On the contrary, breakpoints set at an erroneously low level might lead to abandonment of antimicrobials that are actually effective.

Carbapenems breakpoints for *Acinetobacter* species have been set by several organizations, including the Clinical and Laboratory Standards Institute (CLSI)[7] and the European Committee on Antimicrobial Susceptibility Testing (EUCAST) [8]. There are some discrepancies between the carbapenems breakpoints set by these two major organizations. The current breakpoints determined by CLSI for minimal inhibitory concentrations (MICs) of imipenem and meropenem against *Acinetobacter* species are ≤ 2 mg/L (susceptible), 4 mg/L (intermediate), and ≥ 8 mg/L (resistant) [7]. EUCAST breakpoints for imipenem and meropenem MICs against *Acinetobacter* are ≤ 2 mg/L (susceptible), 4 and 8 mg/L (intermediate), and ≥16 mg/L (resistant) [8]. The major discrepancy among the breakpoints set by these two different organizations is for MIC = 8 mg/L. Moreover, MIC = 4 mg/L is considered as intermediate by both organizations, and the clinical efficacy in this category is uncertain. Although the carbapenems have been used for the treatment of Ab group infection for several decades, to the best of our knowledge, the clinical data supporting the carbapenem breakpoints for Ab group are lacking.

In this retrospective chart review study, we examined the clinical outcome of patients who had received carbapenem therapy for Ab group bacteremia to validate the current carbapenem breakpoints, and to delineate the clinical outcome of patients acquiring isolates with MIC = 4 mg/L and those acquiring isolates with MIC = 8 mg/L. The results would provide important clinical data for the optimization of the current carbapenem breakpoints.

## Materials and Methods

### Study Design and Patients

A retrospective cohort study among adult patients with Ab group bacteremia at Taipei Veterans General Hospital (TVGH), a major tertiary medical center with 2900 beds in Taipei, Taiwan, was undertaken between December 2005 and December 2013. Patients who had monomicrobial growth of Ab group in blood cultures, had initiated either imipenem or meropenem as monotherapy and initial therapy within 24 hours after bacteremia onset, and received a minimum of 24 hours of carbapenem therapy were included. In TVGH, imipenem was given at 500 mg intravenously every 6 hours, and meropenem at 1 g intravenously every 8 hours. The doses of carbapenems were adjusted according to the renal function, as recommended previously [9]. Both the carbapenems were normally infused for 30–60 minutes. Patients < 18 years of age, those who received carbapenem with a dosage inappropriate for end organ function, and those with incomplete medical records were excluded. The protocol was approved by the hospital’s institutional review board (IRB No. 2014-07-006CC). Written informed consent was waived by IRB due to the retrospective nature of the analysis using information contained in medical charts and records, which were anonymized and de-identified prior to analysis.

### Bacterial Identification, Clonal Study, Antimicrobial Susceptibility Testing and Detection of Carbapenemase Genes

The first isolate of the patients was included in the following microbiological studies. The presumptive identification of the isolates to Ab group was determined using the API ID 32GN system (bioMerieux, Marcy l’Etoile, France). A multiplex polymerase chain reaction (PCR) assay was performed to identify *A*. *baumannii* to the genomic species level [10]. Genomic species of isolates recognized as non-*A*. *baumannii* Ab group were identified by 16S–23S ribosomal DNA intergenic spacer sequence analysis as previously described [11]. The clonality was determined by pulsed-field gel electrophoresis as previously described [12]. MICs of carbapenems and susceptibilities of other antimicrobial agents were determined by agar dilution in accordance with the recommendations of CLSI [7]. Multidrug resistance (MDR) was defined as non-susceptibility to at least one agent in three or more following antimicrobial categories: antipseudomonal cephalosporins, antipseudomonal carbapenems, β-lactam/β-lactamase inhibitor combinations, fluoroquinolones, and aminoglycosides.

Detection of the carbapenem-hydrolyzing class D β-lactamase (CHDL) genes (*bla*_OXA-23_-like, *bla*_OXA-24_-like, *bla*_OXA-51_-like, *bla*_OXA-58_-like and *bla*_OXA-143_-like) was performed by multiplex PCR assay [13]. Upstream location of insertion sequences (ISs) (IS*Aba1* upstream of *bla*_OXA-51_-like, *bla*_OXA-23_-like, IS*1008* or IS*1006* upstream of *bla*_OXA-58_-like) was sought by PCR mapping [14–18]. Metallo-β-lactamases genes were detected by PCR assays [15].

### Data Collection

Clinical information was retrieved from medical charts. Patients were assessed for demographic characteristics, comorbidities, duration of hospital and ICU stays, time of receipt, dose and route of therapy with individual antimicrobial drugs, the presence of central venous catheters, an endotracheal tube or tracheostomy, a ventilator, a thoracic drain, or an abdominal drain at the time of bacteremia onset. The onset of bacteremia was defined at the day when then blood culture that eventually yielded Ab group was obtained [19]. Episodes of bloodstream infection were considered acquired in ICU if they appeared 48 hours after ICU admission. Recent stay in ICU was defined as being in ICU within 2 weeks before bacteremia onset. Previous use of antimicrobials was defined as the use of antimicrobials within 30 days before bacteremia onset [19]. Immunosuppressive therapy was defined as immunosuppressive agents use within 2 weeks or corticosteroids use at a dosage equivalent to or higher than 15 mg of prednisolone daily for 1 week within 4 weeks before bacteremia onset. Chemotherapy was defined as receipt of cytotoxic agents within 6 weeks before bacteremia onset. Recent surgery was defined as operations performed within 4 weeks before the onset of bacteremia. The source of bacteremia was determined according to the definitions of the US Centers for Disease Control and Prevention [20]. The severity of patient infection was evaluated using the Acute Physiology and Chronic Health Evaluation (APACHE) II score within 24 hours before bacteremia onset. The all-cause 30-day mortality rate was used as the endpoint, and was defined as death occurring within 30 days after the date of bacteremia onset. For patients who were discharged before the 30-day limit, status was determined by review of outpatient records or by contacting the patient directly. No patients in this group were lost to follow-up.

### Statistical Analysis

Continuous variables were compared with the Mann–Whitney *U* test (for nonnormally distributed variables) or Student *t* test (for normally distributed variables). Categorical variables were evaluated with the χ^2^ test with Yates correction or Fisher’s exact test. The Wilcoxon signed-rank test was used to determine whether there is a statistically significant difference between paired samples. The time to mortality, defined as the interval between bacteremia onset and death, was analyzed using the Kaplan–Meier survival analysis, and the nonparametric (log-rank) test was used to compare survival functions in different groups. Logistic regression models or Cox proportional hazard regression models were used to explore independent prognostic factors associated with 30-day mortality. Univariable analyses were perform separately for each of the risk factor to ascertain the odds ratio (OR) or hazard ratio (HR) and 95% confidence interval (CI). All biologically plausible variables with a *P* value of < 0.20 in the univariable analysis were considered for inclusion in the logistic regression model or Cox regression model in the multivariable analysis. A backward selection process was utilized. A *P* value of < 0.05 was considered statistically significant.

To determine breakpoints, a binary recursive partitioning methodology, classification and regression tree (CART) modeling, was utilized to attempt to define a split between interval variables and outcomes. Specifically, CART was used to identify the breakpoint in the carbapenem MIC distribution that maximized the difference in 30-day mortality, thereby dividing the study population into two groups: those with a high likelihood of 30-day mortality and those with a low likelihood of 30-day mortality. Pruning and 10-fold cross-validation were used in the CART analysis to select the optimal nested subtree with the smallest misclassification cost.

All statistical analyses were performed with the statistical package PASW for Windows version 18 (SPSS, Chicago, IL, USA).

## Results

### Patients and *Acinetobacter* Isolates

During the study period, a total of 786 patients who had monomicrobial Ab group bacteremia and complete medical data were identified. Among 238 patients who received carbapenems (imipenem or meropenem) for treatment of Ab group bacteremia, 117 who started to receive imipenem (53 patients) or meropenem (64 patients) as monotherapy and initial therapy within 24 hours of bacteremia onset and were included. The treatment duration of imipenem and meropenem was 10 ± 7 days and 12 ± 9 days, respectively (*P* = 0.072). After initial carbapenem monotherapy, 38 of the 117 patients were switched to other antimicrobial therapy or treated with other antimicrobial agent in combination with carbapenem (S1 Table). A total of 51 patients who infected with Ab group isolates with carbapenem MIC of ≥ 8 mg/L. Thirty-one patients switched to other regimens alone or in combination or in combination with carbapenem. Ten patients died before the laboratory results came out therefore they did not receive alternative antimicrobial therapy. Ten patients got improved after receiving carbapenem therapy therefore they kept on carbapenem monotherapy. No specific alternative antimicrobial therapy alone or in combination with carbapenem or other antimicrobial agent was associated with significantly increased or decreased 30-day mortality. Seventy-five (75/117, 64.1%) patients had received antimicrobial agents prior to carbapenem therapy. The infections that were treated prior to the *Acinetobacter* infection were caused by *Pseudomonas aeruginosa*, *Stenotrophomonas maltophilia*, *Staphylococcus aureus*, *Escherichia coli*, *Klebsiella pneumoniae*, *Enterobacter*, *Citrobacter*, *Chryseobacterium meningosepticum*, and *Enterococcus*. None was caused by *Acinetobacte*r spp. Most of them (65/75, 86.7%) received antimicrobial agents that were inactive against the causative microorganisms, but it was not associated with increased 30-day mortality. There was no significant difference in survival based on the class of the antimicrobial agent individual patient received prior to carbapenem therapy.

One hundred and seventeen Ab group isolates (one from each patient) were identified as *A*. *baumannii* (66 isolates, 44 clones), *A*. *pitti* (13 isolates, 10 clones) and *A*. *nosocomialis* (38 isolates, 28 clones). The MICs for meropenem or imipenem of Ab group isolates distributed evenly over the 8-year period. The MICs for meropenem or imipenem for the same isolate were not always equal, and there was no significant difference between the MICs for imipenem and those for meropenem (*P* = 0.256). Therefore, only the MIC of the carbapenem that the patient received was presented.

The 30-day mortality rate among patients with *A*. *baumannii*, *A*. *pitti* and *A*. *nosocomialis* bacteremia was similar by survival analysis (S1 Fig). In addition, there was also no significant difference in the 30-day mortality between patients receiving either imipenem or meropenem, as shown by survival analysis (S2 Fig) or by bivariate analysis in different susceptibility categories (including MIC ≤ 2, 4, 8 or ≥16 mg/L). There is only one breakpoint for imipenem and meropenem against all *Acinetobacter* species; therefore, we grouped together the three different Ab group species and two carbapenems in our analysis.

### Comparison among Patients Acquiring Isolates with Different Carbapenem MICs

**Fig 1:**
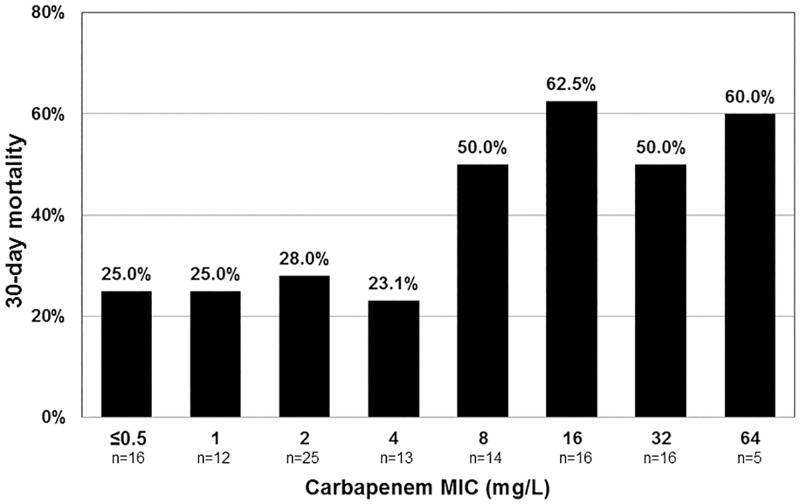
Thirty-day mortality rate of patients with *Acinetobacter baumannii* (Ab) group bacteremia in different susceptibility categories. The rate was significantly lower in those with carbapenem minimal inhibitory concentration (MIC) ≤ 4 mg/L comparing to those with carbapenem MIC ≥ 8 mg/L.

The number of patients/isolates that had carbapenem MIC of < = 0.5, 1, 2, 4, 8, 16, 32, and 64 was shown in Fig 1. The 30-day mortality rates varied by the carbapenem MIC of the Ab group isolate (Fig 1). The mortality rate did not differ significantly between the patients acquiring isolates with MIC ≤ 2 mg/L and 4 mg/L (26.4% vs 23.1%, *P* = 1.000). Then we determined the clinical outcome of patients acquiring isolates with MIC = 8 mg/L (14 patients), which indicated resistance and intermediate susceptibility according to the CLSI and EUCAST guidelines, respectively. The 30-day mortality rate of patients acquiring isolates with MIC = 8 mg/L did not differ significantly from that in patients with MIC ≥16 mg/L isolates (50% vs 56.8%; *P* = 0.906).

**Fig 2:**
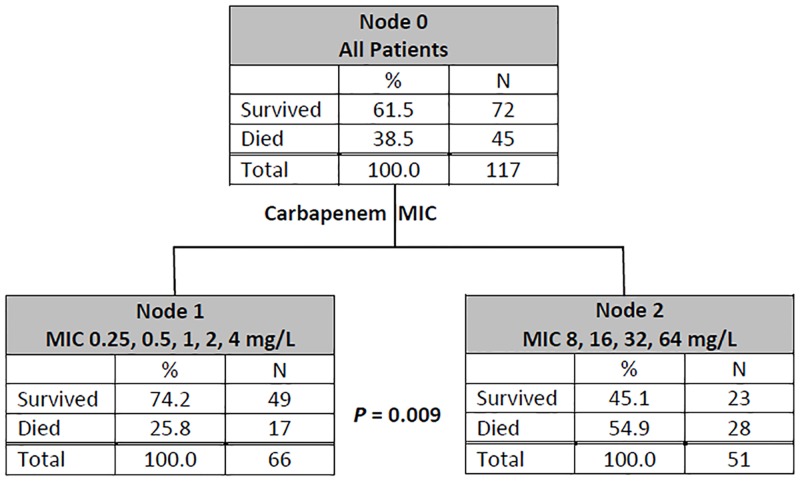
Classification and Regression Tree (CART) analysis determined a split of carbapenem minimal inhibitory concentration (MIC) between 4 and 8 mg/L and predicted differences in mortality.

**Fig 3:**
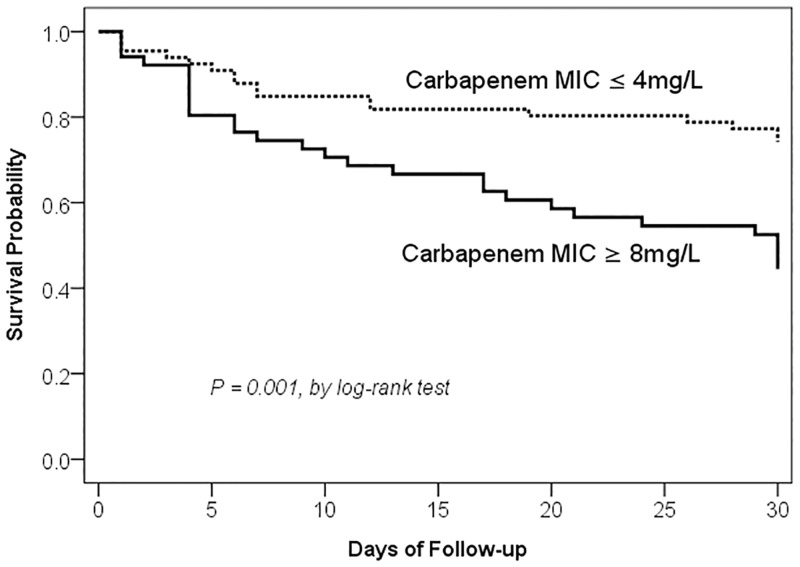
Comparison of Kaplan–Meier survival curves, at 30 days, between patients with *Acinetobacter baumannii* group bacteremia caused by isolates having minimal inhibitory concentration (MIC) ≤ 4 mg/L or ≥ 8 mg/L.

The carbapenem MIC breakpoint that maximized the difference in 30-day mortality was determined by CART analysis and was found between carbapenem MICs of ≤ 4 mg/L and ≥ 8 mg/L (Fig 2). Patients acquiring Ab group isolates with carbapenem MIC ≥8 mg/L had a significantly higher 30-day mortality than those acquiring isolates with MIC ≤ 4 mg/L by bivariate analysis (54.9% [28/51] vs 25.8% [17/66]; *P* = 0.003). The Kaplan-Meier survival analysis also revealed that the 30-day mortality rate was significantly higher in patients acquiring Ab group isolates with carbapenem MIC ≥ 8 mg/L than in those acquiring isolates with MIC ≤ 4 mg/L (Fig 3).

Patients were accordingly stratified to carbapenem MIC ≤ 4 mg/L and ≥ 8 mg/L with baseline demographics, clinical characteristics, and microbiologic characteristics presented in Table 1. There were no significant differences of Charlson cormobidity index and APACHE II scores at bacteremia onset between patients acquiring isolates with carbapenem MIC ≤ 4 mg/L and ≥ 8 mg/L (Table 1). Nevertheless, there were different characteristics between the two groups of patients that might have affected patient outcome. A multivariate logistic regression analysis was performed to identify whether acquisition of isolates with MIC ≥ 8 mg/L was independently associated with 30-day mortality (Table 2). It revealed that acquisition of Ab group isolates with carbapenem MIC ≥8 mg/L, shock at bacteremia onset, and higher APACHE II score at bacteremia onset were independently associated with 30-day mortality. Acquisition of isolates with carbapenem MIC ≥8 mg/L remained an independent predictor of 30-day mortality by Cox regression analysis (Table 3).

**Table 1:** Univariate comparison between patients acquiring *Acinetobacter baumannii* group with carbapenem MIC ≤ 4mg/L and MIC ≥ 8mg/L.

	Carbapenem MIC ≤ 4mg/L (*n* = 66)	Carbapenem MIC ≥ 8mg/L (*n* = 51)	*P*-value
Demographic characteristics			
Age, median (IQR), years	77.5 (64.5–82.25)	78 (64–82)	0.987
Male sex, No. (%)	52/14 (78.8/21.2)	37/14 (72.5/27.5)	0.572
Acquired in ICU, No. (%)	38 (57.6)	40 (78.4)	0.030
Recent stay in ICU	41 (62.1)	44 (86.3)	0.007
Length of hospitalization before bacteremia, median (IQR), days	19.5 (10–45.25)	27 (17–52)	0.122
Previous use of antibiotics, No. (%)	56 (84.8)	49 (96.1)	0.093
Comorbid condition, No. (%)			
Alcoholism	7 (10.6)	4 (7.8)	0.754
Liver cirrhosis	1 (1.5)	1 (2.0)	1.000
Chronic obstructive pulmonary disease	14 (21.2)	13 (25.5)	0.746
Chronic kidney disease 16 (31.4) 1.000	20 (30.3)	16 (31.4)	1.000
Type 2 diabetes mellitus 17 (33.3) 1.000	23 (34.8)	17 (33.3)	1.000
Hypertension	25 (37.9)	13 (25.5)	0.223
Coronary artery disease	11(16.7)	15 (29.4)	0.119
Congestive heart failure	6 (9.1)	9 (17.6)	0.274
Cerebrovascular accident	16 (24.2)	10 (19.6)	0.709
Collagen vascular disease	6 (9.1)	4 (7.8)	1.000
Immunosuppressant therapy	6 (9.1)	9 (17.6)	0.274
Solid tumor	16 (24.2)	12 (23.5)	1.000
Hematological malignancy	5 (7.6)	4 (7.8)	1.000
Chemotherapy	8 (12.1)	4 (7.8)	0.653
Neutropenia	1 (1.5)	3 (5.9)	0.316
Trauma	1 (1.5)	0 (0)	1.000
Recent surgery	22 (33.3)	22 (43.1)	0.372
Charlson comorbidity index, median (IQR)	7 (5–9)	8 (6–9)	0.139
Invasive Procedures, No. (%)			
Central venous catheter	47 (71.2)	46 (90.2)	0.022
Endotracheal intubation or tracheostomy	50 (75.8)	46 (90.2)	0.076
Ventilator use	51 (77.3)	46 (90.2)	0.111
Hemodialysis	6 (9.1)	11 (21.6)	0.102
Thoracic drain	4 (6.1)	7 (13.7)	0.206
Abdominal drain	5 (7.6)	8 (15.7)	0.277
Sources of bacteremia, No. (%)			
Pneumonia	45 (68.2)	37 (72.5)	0.758
Catheter	5 (7.6)	4 (7.8)	1.000
Urinary tract infection	4 (6.1)	1 (2.0)	0.385
Intra-abdominal infection	5 (7.6)	1 (2.0)	0.230
Wound	3 (4.5)	3 (5.9)	1.000
Primary bacteremia	7 (10.6)	6 (11.8)	1.000
Regimen of therapy			
Imipenem	32 (48.5)	21 (41.2)	0.548
Meropenem	34 (51.5)	30 (58.8)	0.548
Duration of carbapenem therapy, median (IQR), days	14 (6–15)	8 (4–15)	0.139
Outcome			
Shock, No. (%)	30 (45.5)	19 (37.3)	0.482
APACHE II score, median (IQR)	26 (21.75–32)	30 (24–34)	0.093
30-day mortality, No. (%)	17 (25.8)	28 (54.9)	0.003
Length of stay after bacteremia for survivors, median (IQR), days	34 (21–64)	30 (16–41)	0.418
Bacterial species of causative micro-organisms, No. (%)			
Bacteremia due to *A*. *baumannii*	39 (59.1)	27 (52.9)	0.633
Bacteremia due to *A*. *nosocomialis*	19 (28.8)	19 (37.3)	0.441
Bacteremia due to *A*. *pittii*	8 (12.1)	5 (9.8)	0.921
Microbiological characteristics of causative micro-organisms, No. (%)			
Multidrug resistance	36 (54.5)	39 (76.5)	0.024
Isolates harboring IS*Aba1*-*bla*_OXA-51_-like	14 (21.2)	14 (29.4)	0.422
Isolates harboring IS*Aba1*-*bla*_OXA-23_-like	1 (1.5)	9 (17.6)	0.002
Isolates harboring IS*1008*-ΔIS*Aba3*-*bla*_OXA-58_-like	1 (1.5)	8 (15.7)	0.010
Isolates harboring *bla*_OXA-24_-like	1 (1.5)	1 (2.0)	1.000
Isolates harboring *bla*_IMP_-like	0 (0)	4 (7.8)	0.034
Isolates harboring *bla*_VIM_-like	1 (1.5)	8 (15.7)	0.010

IQR, interquartile range; MIC, minimal inhibitory concentration; ICU, intensive care unit; APACHE II, Acute Physiology and Chronic Health Evaluation II.

**Table 2:** Logistic regression analysis of prognostic factors associated with 30-day mortality among patients treated with carbapenem for *Acinetobacter baumannii* group bacteremia.

Variables	Survivors (*n* = 72)	Non -survivors (*n* = 45)	Univariate analysis		Multivariate analysis	
			OR (95% CI)	*P*	OR (95% CI)	*P*
Recent stay in ICU	48 (66.7)	37 (88.2)	2.312 (0.933–5.732)	0.070		
Shock	20 (27.8)	29 (64.4)	4.712 (2.119–10.478)	<0.001	6.798 (2.583–17.893)	<0.001
Presence of central venous catheter	54 (75.0)	39 (86.7)	2.167 (0.788–5.958)	0.134		
Respiratory tract as source of infection	46 (63.9)	36 (80.0)	2.261 (0.943–5.421)	0.068		
APACHE II score	26 (21–30.75)	30 (25–37)	1.088 (1.032–1.146)	0.002	1.072 (1.014–1.134)	0.015
Acquisition of isolates with MIC ≥8 mg/L	23 (31.9)	28 (62.2)	3.509 (1.608–7.656)	0.002	5.125 (1.946–13.498)	0.001

OR, odds ratio; CI, confidence interval; ICU, intensive care unit; APACHE II, Acute Physiology and Chronic Health Evaluation II; MIC, minimal inhibitory concentration

**Table 3:** Cox regression analysis of prognostic factors associated with 30-day mortality among patients treated with carbapenem for *Acinetobacter baumannii* group bacteremia.

Variables	Univariate analysis		Multivariate analysis	
	HR (95% CI)	*P*	HR (95% CI)	*P*
Recent stay in ICU	1.922 (0.895–4.129)	0.094		
Shock	3.222 (1.746–5.947)	<0.001	3.107 (1.678–5.753)	<0.001
Presence of central venous catheter	1.880 (0.796–4.443)	0.150		
Presence of endotracheal tube or tracheostomy	1.924 (0.759–4.875)	0.168		
Presence of thoracic drainage	2.104 (0.939–4.718)	0.071		
Presence of abdominal drainage	1.827 (0.815–4.098)	0.144		
Respiratory tract as source of infection	1.911 (0.920–3.968)	0.082		
APACHE II score	1.071 (1.030–1.114)	0.001	1.059 (1.016–1.104)	0.007
Acquisition of isolates with MIC ≥8 mg/L	2.560 (1.399–4.685)	0.002	2.630 (1.431–4.834)	0.002

HR, hazard ratio; CI, confidence interval; ICU, intensive care unit; APACHE II, Acute Physiology and Chronic Health Evaluation II; MIC, minimal inhibitory concentration

### Risk Factors for Acquisition of Isolates with MIC ≥8 mg/L

In the univariate analysis, patients acquiring isolates with MIC ≥8 mg/L were more likely to have had acquisition of the isolate in ICU, a recent stay in ICU, and presence of central venous catheters at bacteremia onset (Table 1). Comparing with the bloodstream isolates with carbapenem MIC ≤ 4 mg/L, those with carbapenem MIC ≥ 8 mg/L had a significantly greater rate of multidrug resistance and were more likely to carry carbapenemase gene associated genetic structures such as IS*Aba1*-*bla*_OXA-23_-like, IS*1008* (or IS*1006*)-ΔIS*Aba3*-*bla*_OXA-58_-like, *bla*_IMP_-like and *bla*_VIM_-like (Table 1). Among the 51 isolates with a carbapenem MIC ≥ 8 mg/L, the carbapenemase genes and associated insertion sequences carried were *bla*_OXA-51_-like with upstream IS*Aba1* (IS*Aba1*-*bla*_OXA-51_-like, 14 isolates), *bla*_OXA-23_-like with upstream IS*Aba1* (IS*Aba1*-*bla*_OXA-23_-like, 9 isolates) *bla*_OXA-58_-like with upstream IS*Aba3* truncated by IS*1008* or IS*1006* (IS*1008* [or IS*1006*]-ΔIS*Aba3*-*bla*_OXA-58_-like, 9 isolates), *bla*_OXA-24_-like (1 isolate), *bla*_IMP_-like (4 isolates) or *bla*_VIM_-like (8 isolates). Seven isolates with a carbapenem MIC ≥ 8 mg/L did not carry any currently known carbapenemase gene. Multivariate analysis revealed that the only factor that could independently predict acquisition of Ab group isolates with carbapenem MIC ≥ 8 mg/L was recent stay in ICU (OR, 3.845; 95% CI, 1.485–9.956; *P* = 0.006).

## Discussion

This retrospective study provided the clinical data to support that carbapenem MIC ≤ 4 mg/L represents susceptibility and MIC ≥ 8 mg/L resistance in patients with Ab group bacteremia. Acquisition of isolates with MIC ≥ 8 mg/L was independently associated with poor outcome in patients receiving carbapenem therapy for Ab group bacteremia. The Ab group isolates with a high MIC carried different carbapenemase genes and belonged to different clones, suggesting that our results were not applicable only to specific *Acinetobacter* clones or isolates with specific resistance mechanisms.

Outcome data from clinical studies are essential to optimize or harmonize the antimicrobial breakpoints, especially when there are discrepancies between breakpoints set by different organizations. The susceptibility breakpoints are determined after integration of the data from MIC distribution of pathogens, pharmacokinetics/pharmacodynamics (PK/PD) and clinical studies [21, 22]. The former two data types for setting carbapenem breakpoints for *Acinetobacter* species have been addressed [23–26], but to the best of our knowledge, our present results provide the first clinical data which helps to confirm the carbapenem breakpoints in patients with *Acinetobacter* infection.

Although the best clinical data for setting breakpoints are those derived from randomized control trials, it is hard to perform such trials for many reasons [22, 27]. Most importantly, it is not ethical to perform a trial including patients infected by isolates with MICs within the resistance range where therapy is likely to fail.

Breakpoints proposed by different organizations can vary according to the use of different predefined pharmacodynamic targets (PDTs), even when applying similar PK/PD simulations. For carbapenems, the PDT is the percentage of the dosing interval for which the free drug concentration exceeds the MIC (% *f*T >MIC) [23, 24]. The optimal PDT for *Acinetobacter* is undetermined. A PK/PD simulation using % *f*T >30% as PDT has proposed that the susceptibility breakpoints for both imipenem and meropenem for *A*. *baumannii* are ≤ 4 mg/L [23, 24]. In this study, the mortality rate did not differ significantly between the patients acquiring isolates with carbapenem MIC ≤ 2 mg/L and 4 mg/L, but patients acquiring Ab group isolates with carbapenem MIC ≥8 mg/L had a significantly higher 30-day mortality than those acquiring isolates with MIC ≤ 4 mg/L, providing clinical data to support that MIC ≤ 4 mg/L can be considered as carbapenem susceptibility.

The therapy of patients infected by isolates with intermediate antimicrobial susceptibility might be effective if the antimicrobial agents are concentrated at site of infection or used at higher dose [22]. In the present study, it was clear that the mortality rate of patients with Ab group bacteremia caused by isolates with carbapenem MIC 8 mg/L was as high as that with carbapenem MIC ≥ 16 mg/L, if the patients received the commonly used dose of imipenem or meropenem. Hence, for Ab group bacteremia, carbapenem MIC of 8 mg/L should be regarded as resistant.

Identification of patients acquiring isolates with MIC ≥8 mg/L is important because these patients are likely to fail carbapenem therapy. For institutes that are able to perform PCR before the MIC data are available, the detection of the genetic structures IS*Aba1*-*bla*_OXA-23_-like, IS*1008*(or IS*1006)*-ΔIS*Aba3* -*bla*_OXA-58_-like, *bla*_IMP_-like and *bla*_VIM_-like in an Ab group isolate can be used as a resistance marker to predict higher carbapenem MIC. If the PCR is not feasible, recent stay in ICU is an independent factor associated with acquisition of isolates with carbapenem MIC ≥ 8 mg/L.

The limitations of the study are only one center (management may be different than in others and this impact on outcome), long study period (changes in management not considered), and drug levels were not measured. However, the study was strengthened by the inclusion of a large number of patients acquiring different clones of Ab group isolates with various carbapenem susceptibilities, with receipt of carbapenem as initial and monotherapy for Ab group bacteremia, and a well-defined end point of 30-day mortality.

In conclusion, this study provided the clinical data supporting carbapenem breakpoints for Ab group in patients with bacteremia. Our results suggested that MIC ≤ 4 mg/L can be considered as antimicrobial susceptibility and ≥ 8 mg/L as resistance in patients with Ab group bacteremia.

## Supporting Information

S1 Fig Comparison of Kaplan–Meier survival curves, at 30 days, between patients with bacteremia caused by either *A*. *baumannii*, *A*. *nosocomialis* or *A*. *pitti*, which was analysed in a whole population (A) or subpopulation having isolates with MIC ≤ 4 mg/L (B) or ≥ 8 mg/L (C). (PDF)

S2 Fig Comparison of Kaplan–Meier survival curves, at 30 days, between patients treated with either imipenem or meropenem for their *Acinetobacter baumannii* group bacteremia. (TIF)

S1 Table Clinical information of patients with *Acinetobacter baumannii* group bacteremia who received alternative antimicrobial therapy after initial carbapenem monotherapy. (PDF)
