# Supplementary figures and images for: Carbapenem Breakpoints for Acinetobacter baumannii Group: Supporting Clinical Outcome Data from Patients with Bacteremia
Source: PLoS One. 2016 Sep 19;11(9):e0163271. doi: 10.1371/journal.pone.0163271 (PMC5028070; doi:10.1371/journal.pone.0163271)

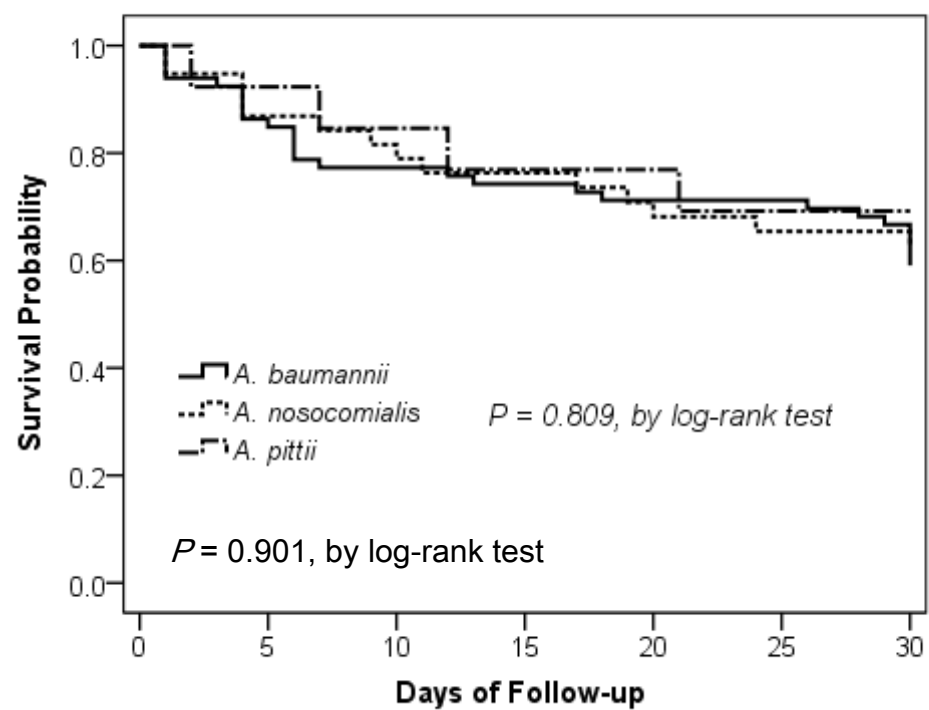

S1 Fig. (A)

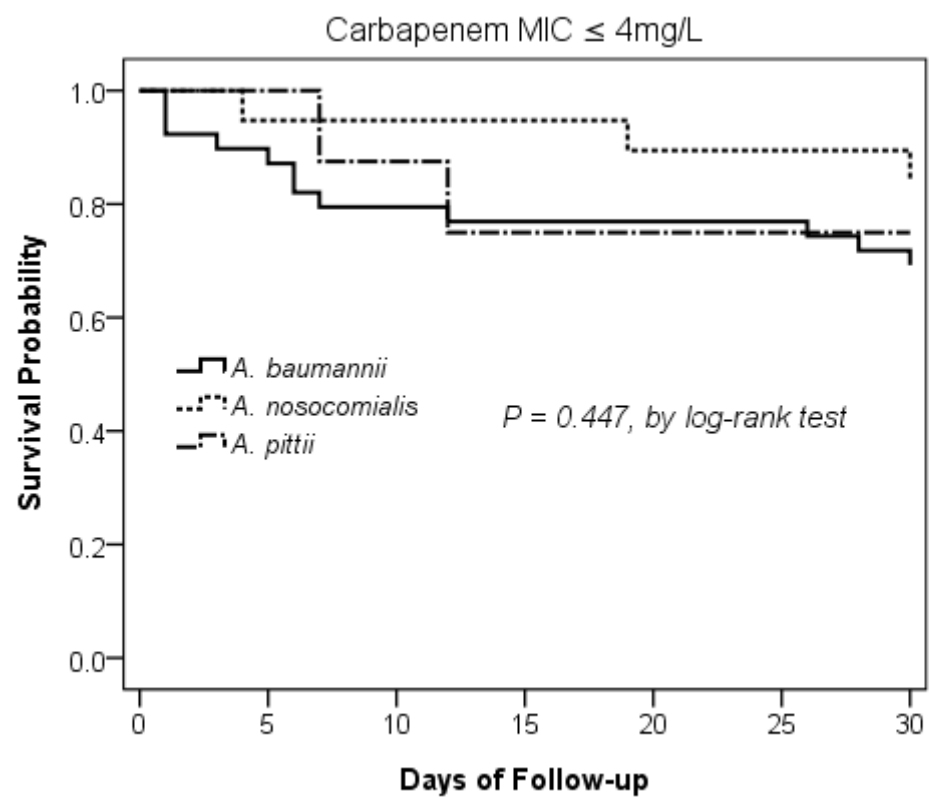

S1 Fig. (B)

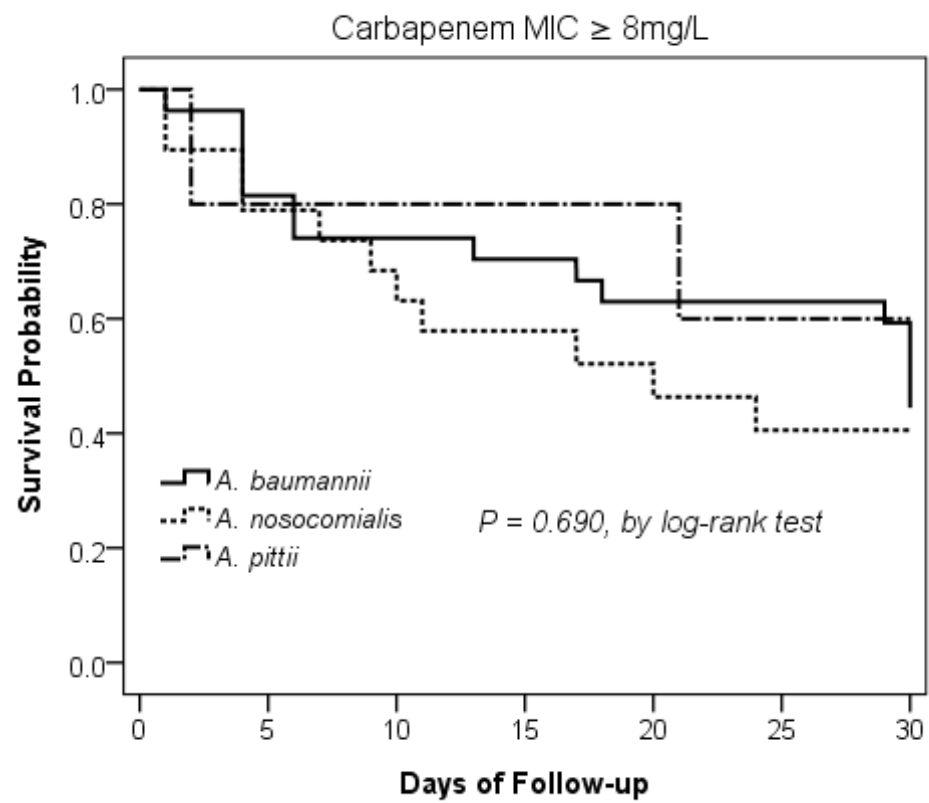

S1 Fig. (C)

Supplement: S1 Fig — (PDF) [file pone.0163271.s001.pdf]

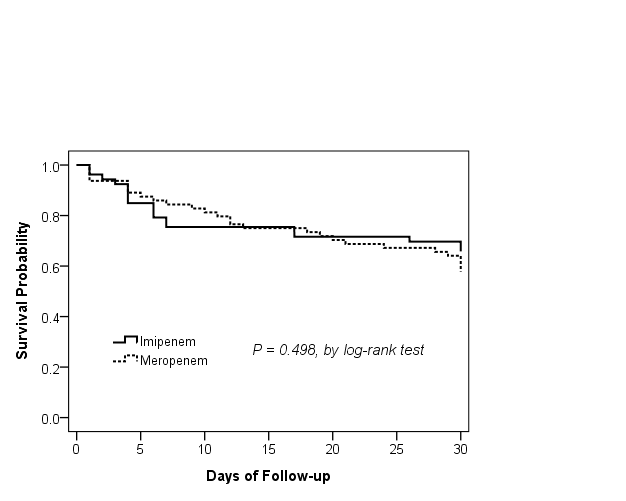

Supplement: S2 Fig — (TIF) [file pone.0163271.s002.tif]
