# Supplementary material for: Carbapenem Breakpoints for Acinetobacter baumannii Group: Supporting Clinical Outcome Data from Patients with Bacteremia
Source: PLoS One. 2016 Sep 19;11(9):e0163271. doi: 10.1371/journal.pone.0163271 (PMC5028070; doi:10.1371/journal.pone.0163271)
Supplement: S1 Table — (PDF) [file pone.0163271.s003.pdf]

Patient No.	Carbapenem MIC (mg/L)	Duration of initial carbapenem monotherapy (days)	Antimicrobial therapy switched to	Appropriateness of alternative therapy	APACHE II score at bacteremia onset	30-day mortality
1	4	5	Ciprofloxacin for 14 days	No	26	No
2	0.5	4	Ciprofloxacin + Ampicillin/sulbactam for 4 days	No	40	Yes
3	32	2	Cefepime for 1 day	Yes	30	Yes
4	8	3	Ceftazidime for 9 days	Yes	19	Yes
5	8	4	Ciprofloxacin for 10 days	No	29	No
6	2	3	Piperacillin/tazobactam for 12 days	Yes	20	No
7	2	1	Ampicillin/sulbactam for 7 days	Yes	20	No
8	16	4	Tigecycline for 9 days	No	12	No
9	16	3	Colistin for 4 days	Yes	38	Yes
10	4	4	Meropenem + sulbactam for 8 days	Yes	26	No
11	16	4	Cefepime + sulbactam for 10 days	No	9	No
12	32	3	Meropenem + tigecycline for 12 days	Yes	18	No
13	16	4	Tigecycline + ciprofloxacin for 8 days	Yes	33	Yes
14	2	3	Cefepime + sulbactam for 14 days	Yes	32	No
15	1	2	Imipenem + sulbactam for 9 days	Yes	26	No
